# Post-Laminectomy Wound Infections: Colonized Seromas Mimicking Wound Infections

**DOI:** 10.3390/jcm3010191

**Published:** 2014-02-14

**Authors:** Burke A. Cunha, Eileen D. Abruzzo, Paul E. Schoch

**Affiliations:** 1 Infection Control Department, Clinical Microbiology Laboratory and Infectious Disease Division, Winthrop-University Hospital, Mineola, NY 11051, USA; E-Mails: eabruzzo@winthrop.org (E.D.A.); pschoch@winthrop.org (P.E.S.); 2 School of Medicine, State University of New York, Stony Brook, NY 11051, USA

**Keywords:** mimics of wound infections, colonized seromas, post-operative fever, prophylactic antibiotics, wound colonization *vs.* infection

## Abstract

Objective: Post-operative laminectomy wounds are frequently accompanied by seromas. Post-operative wound drainage may be colonized or infected. The differentiation of wound colonization from infection is difficult for non-infectious disease physicians. Methods: External chart reviewers classified 31/1531 laminectomies (over three years) as post-operative wound infections. We re-evaluated these cases using infectious disease criteria, *i.e.*, while pathogens may be cultured from both colonized and infected wounds, only wound infections have a purulent discharge with abundant white blood cells (WBCs) on Gram stain. Colonized wounds have positive wound cultures but no/few WBCs on Gram stain. Results: We found only 11/31 actual wound infections, the remainder were not *bona fide* wound infections, but were colonized seromas. Conclusion: Post-laminectomy colonized seromas that are culture positive for one or more organisms often mimic wound infections. In the era of public reporting of nosocomial infections, it is important that external reviewers differentiate colonization from infection to provide regulatory agencies with accurate data.

## 1. Introduction

Laminectomies are among the most complicated orthopedic/neurosurgical procedures. Complicated surgical spine procedures are technically difficult and are of long duration which increases the potential for infectious complications, *i.e.*, deep wound infections [1,2,3,4]. Post-operative laminectomy wound infections may be due to several procedure related factors, e.g., procedure duration/complexity and may be due to host related factors, e.g., age, weight, diabetes and host defense status. With a good aseptic surgical technique, prophylactic antibiotics are given to minimize post operative wound infections [5,6,7,8,9]. We became interested in clinically differentiating colonized from infected seromas, which are a particular problem in post-laminectomy surgery, to avoid needless antibiotic therapy and to accurately classify these wounds.

## 2. Materials and Methods

During the past three years (2010–2012) a total of 1531 laminectomies were performed in our 600 bed university-affiliated teaching hospital. Non-infection control (IC) and non-infectious disease (ID) external chart reviewers reported a relatively high incidence of wound infections post-laminectomy. Since the number of cases seemed unusually high, we re-reviewed 31 cases classified as post-laminectomy wound infections to determine the number of actual infections. In our review, the duration of the case, age, weight, comorbidities, history of previous infections, individual surgeons, laminectomy location and pre-operative antibiotic regimens were analyzed. In addition, microbiological data were correlated with the clinical findings and pre-operative antibiotic regimens.

## 3. Results

Thirty one potential post-laminectomy wound infections identified over a three year period were reviewed; of these only 11 cases met infectious disease criteria for wound infections (Table 1) [10]. Among the 11 post-laminectomy wound infections, we found no procedure or individual surgeon related common denominators. Furthermore, a variety of wound isolates were identified; *Klebsiella pneumoniae*, methicillin resistant *Staphylococcus aureus* (MRSA), methicillin sensitive *Staphylococcus aureus* (MSSA), coagulase negative staphylococci (CoNS), and *E. coli*. There was no relationship between the spectrum of the prophylactic antibiotics used and organism cultured from colonized or infected wounds (Table 2, Table 3 and Table 4).

**Table 1:** Winthrop-university hospital, recent experience with potential post-laminectomy wound infections.

Year	Total Cases (1531)	Presumed Wound Infections (31)	Verified Wound Infections (11/31)	Colonized Seromas Not Wound Infections (20/31)
2010	562	1	0	1
2011	530	16	7	9
2012	439	14	4	10

**Table 2:** Potential post-laminectomy wound infections (2010–2012).

Wound Infections	Non-Wound Infections
Cellulitis/Abscess (11/31)	Seromas (20/31)
**Pre-Operative Prophylactic Antibiotics**	
Cefazolin 8/11 (73%)	Cefazolin 16/20 (80%)
Clindamycin 1/11 (9%)	Clindamycin 3/20 (15%)
Vancomycin 1/11 (9%) (plus gentamicin)	Vancomycin 0/20 (0%)
Unknown 1/11 (9%)	Unknown 1/20 (5%)
**The Relationship between Pre-Operative Antibiotics and Post-Operative Wound Cultures**	
**Cefazolin **(8/11)	**Cefazolin**
associated isolates *:	associated isolates *:
MSSA = 3	CoNS = 6
MRSA = 2	MSSA = 1
*M. fortuitum* = 1	VSE = 1
Unknown = 2	*E. coli* = 2
**Clindamycin**	*Proteus* sp. = 1
associated isolates:	Unknown = 2
*E. coli* = 7	Culture negative = 7
**Vancomycin (1/11) (plus gentamicin)**	**Clindamycin**
associated isolates:	associated isolates:
*K. pneumoniae* = 2	CoNS = 2
	Culture negative = 2

* Some cases had multiple isolates; MSSA = methicillin sensitive *Staphylococcus aureu*; MRSA = methicillin resistant *Staphylococcus aureus*; VRE = vancomycin resistant enterococci; CoNS = coagulase negative staphylocci.

**Table 3:** Differential diagnostic features of post-operative laminectomy wounds: colonization *vs.* infection *.

Wound Discharge		Wound Gram Stain		Wound Culture		Infectious Disease Diagnosis
Clear	with	None, few or some WBCs	with	+	→	Colonization
Serous	with	Few or some WBCs	with	+	→	Colonization
Serosanguineous	with	Few or some WBCs	with	+	→	Colonization
Purulent	with	None, few, or some WBCs	with	+	→	Inflammation
Purulent	with	Abundant WBCs	with	+	→	Infection

* Any post-operative patient may have fever, leukocytosis, wound erythema/induration not related to wound infection. WBCs = white blood cells.

**Table 4:** Skin/Wound organisms that should be considered as commensals/colonizers ^†^.

Gram Positive Organisms	Other Organisms	Gram Negative Organisms
Non-Purulent Wound Exudate with a Gram Stain with WBCs		
*Corynebacterium* sp.	Atypical mycobacteria	*Burkholderia cepacia*
*Propionibacterium* sp.		*Stenotrophomonas maltophilia*
*Bacillus* sp.		*Citrobacter* sp.
Group D enterococci		*Acinetobacter* sp.
(VSE/VRE)		*Alcaligenes xyloxidans*
Viridans streptococci		*P. aeruginosa *^†^
Coagulase negative staphylococci		*Klebsiella sp. *^†^
(CoNS)		*Enterobacter* sp. ^†^
MRSA ^†^		*Serratia marcescens *^†^
MSSA ^†^		*Proteus sp.*

^†^ Considered a *bona fide* infection only if a purulent wound exudate has abundant WBCs on Gram stain.

## 4. Discussion

Skin colonization is the rule in the microbial milieu of the hospital. Seromas are common post-laminectomy and positive wound or seroma cultures often represent colonization by skin organisms. Non-infection control and non-infectious disease external chart reviewers, who evaluated 31 patients, were often confused by discordant seroma fluid microbiology results, *i.e.*, Gram stain (number of white blood cells (WBCs) and organisms) and wound/fluid culture results.

The most common cause of misdiagnosis of post-operative laminectomy wound infections was colonized seromas. External chart reviewers (non-infection control and non-infectious disease personnel) had difficulties in correlating clinical and microbiologic data to diagnose or rule out post-laminectomy wound infections. Post-operatively, the skin surrounding incisions may be erythematous, but unless infected is not warm or tender. Furthermore, unlike non-laminectomy post-operative wound infections, laminectomies were commonly complicated by seromas. Colonized seromas draining through the wound may mimic wound infection, but seroma fluid is clear or serosanguineous, but not purulent.

Colonization of wounds and seromas is common but is not preventable by utilizing pre-operative prophylactic antibiotics. Wound Gram stains demonstrating few/no polymorphonuclear cells (PMNs) with positive wound cultures from a body fluid without the clinical criteria of infection are not indicative of wound infection. When the signs of infection are absent, e.g., no surrounding skin erythema, warmth and tenderness, microorganisms cultured from non-purulent seroma fluid indicate colonization but not infection.

## 5. Conclusions

Fever also caused diagnostic confusion for external reviewers in attributing fever/leukocytosis in post-laminectomy patients with positive wound cultures to wound infection. However, post-laminectomy patients often have low-grade fevers due to a variety of other non-wound related infectious or non-infectious causes, e.g., inflammation, atelectasis, phlebitis. In our experience, external chart reviewers have difficulty in interpreting and correlating wound cultures results with microbiologic results, *i.e.*, wound culture *vs.* wound Gram stains to differentiate post-laminectomy wound infections from colonized seromas.
